# Broad H3K4me3 as A Novel Epigenetic Signature for Normal Development and Disease

**DOI:** 10.1016/j.gpb.2016.09.001

**Published:** 2016-10-12

**Authors:** Jie Lv, Kaifu Chen

**Affiliations:** 1 Center for Cardiovascular Regeneration, Department of Cardiovascular Sciences, The Methodist Hospital Research Institute, Houston, TX 77030, USA; 2 Weill Cornell Medical College, Cornell University, New York, NY 10065, USA

The breadth of the enrichment site for post-translational trimethylation of histone H3 at lysine 4 (H3K4me3) on chromatin has attracted great attention recently. H3K4me3, an extensively-studied histone modification, is reported to promote gene transcription by directing preinitiation complex assembly through interaction with effector proteins, *e.g.*, the transcription factor IID (TFIID) complex [1]. Scientists have been interested in the biological implications of signal density, but know little about the importance of the breadth of an H3K4me3 enrichment site. H3K4me3 is usually restricted within a narrow genomic region of approximately one thousand base pairs, with high signal density around the transcription start site (TSS) [2]. However, several groups recently revealed that this is not always the case. A considerable number of H3K4me3 enrichment sites can each be broad while having low signal density. Such broad H3K4me3 domains each spans a genomic region of at least several thousands of base pairs [3], [4], [5], [6], [7]. H3K4me3 domain that is as wide as hundreds of thousands of base pairs was reproducibly observed. The upper limit of H3K4me3 domain width is not clear yet. Results from multiple cell types in different species consistently provide strong evidence showing that H3K4me3 breadth has its own biological implications. These findings suggest that the breadth of H3K4me3 is an unexplored functional epigenetic signal.

## Broad H3K4me3 is associated with cell identity specification and tumor suppression

The broad pattern of H3K4me3 was observed early at some important genes in several cell types [8], [9], [10]. These observations were, at that time, mainly considered exceptions to the general sharp, narrow peak of H3k4me3, and were not expected to have large-scale functional relevance. However, three recent studies systemically investigated H3K4me3 landscape through integrative analysis of large dataset, and both independently revealed that the breadth of enrichment site plays a key role in determining the functions of H3K4me3 [3], [4], [11]. Benayoun et al. [3] performed a large-scale meta-analysis of H3K4me3 and observed a widespread pattern of broad H3K4me3 in multiple cell lines originating from different species. They demonstrated that genes marked with the top 5% of the broadest H3K4me3 domains are enriched in cell type-specific functions in a given cell type and across species. Both single cell and cell population datasets demonstrate that broad H3K4me3 domains are associated with increased transcriptional consistence. Chen et al. [4] systematically studied the epigenetic signatures of cancer driver genes by conducting a large-scale integrative analysis of 1134 genome-wide epigenetic profiles, cancer somatic mutations from >8200 tumor–normal pairs, and experimental data from clinical samples. They identified broad H3K4me3 as the first epigenetic signature for tumor suppressor genes in normal cells. Their data show that broad H3K4me3 peaks, but not the typical H3K4me3 peaks, are associated with increased transcription elongation and enhancer activity. Chen et al. [4] also reported a positive correlation between the broad H3K4me3 and high expression of the associated genes. Dincer et al. [11] specifically analyzed H3K4me3 in the human brain, and revealed that the broadest H3K4me3 domains are associated with synaptic signaling, neuronal functions, and developmental disease mechanisms. In agreement with each other, Benayoun et al. [3], Chen et al. [4], and Dincer et al. [11] all independently revealed that cell type-specific broad H3K4me3 domains are associated with cell identity genes, albeit using different datasets and methodologies. These studies highlighted for the first time the importance of breadth in deciphering the H3K4me3 signal.

## Broad H3K4me3 is reprogramed dramatically during pre-implantation embryonic development

Three more recently published studies systematically analyzed the specific dynamics of broad H3K4me3 in the developing gamete and pre-implantation embryo, indicating their crucial function in regulating gene expression at early developmental stages [5], [6], [7]. Epigenome is dramatically reprogramed after fertilization and during early embryonic development. However, the genome-wide profiling of regions undergoing epigenetic reprograming remains unexplored, largely due to the scarcity of cells from early developmental stages. These studies addressed this technical challenge by developing or improving highly-sensitive and low-input chromatin immunoprecipitation-sequencing (ChIP-Seq) technologies, with some differences between studies. These low-input ChIP-Seq technologies enabled the analysis of histone modifications in as few as 200 cells. Taking advantage of low-input ChIP-Seq, several histone modifications, including H3K4me3, H3K27me3, and H3K27ac, were profiled on a genome-wide scale in cells from early developmental stages. One consistent pattern revealed in all three studies is that both the shape and distribution of H3K4me3 enrichment sites are quite distinct in cells of early developmental stages when compared to embryonic stem cells (ESCs) or mature cell types. Specifically, in early cells (gametes, zygotes, and embryos prior to the 2-cell stage) H3K4me3 marks usually spread across large genomic regions, and each spans more than ten thousand base pairs, with low signal density and large distance from TSS. This unique pattern of H3K4me3 is termed either broad H3K4me3 or non-canonical H3K4me3, to be distinguished from the narrowly-disposed H3K4me3 around the TSS in ES cells and other mature cell types.

Liu et al. [7] used the low-input ChIP-Seq to profile genome-wide reprograming of histone modifications H3K4me3 and H3K27me3 during pre-implantation developmental stages. The authors mapped the two modifications in cells from metaphase II (MII) oocytes to pre-implantation embryos at 2-cell, 4-cell, 8-cell, and morula stages, as well as the inner cell mass (ICM), trophectoderm (TE), ESCs, and trophoblast stem cells (TSCs). They observed an increase in the number of genes marked by broad H3K4me3 from 1783 at 2-cell stage to 5747 and 5932 in ICM and TE, respectively. The number decreases to 1315 and 1428 in ESCs and TSCs, respectively. Notably, their method detected only 12 genes associated with broad H3K4me3 in metaphase II (MII) oocytes. Accordingly, by knocking down the genes encoding histone demethylases, *e.g.*, lysine (K)-specific demethylase 5B (KDM5B), to impede the shortening of broad H3K4me3, the blastocyst formation, but not the development to the morula, was impaired. The authors demonstrated that the high dynamic of broad H3K4me3 occurred mainly between broad and medium status, and barely between broad and narrow status. In agreement with the observation by Chen et al. [4], Liu et al. [7] observed a strong positive correlation between H3K4me3 breadth and RNA expression. Taken together, the authors suggested that the dynamics of H3K4me3 breadth might be a novel mechanism of epigenetic regulation in early cleavage embryos.

Employing similar strategies independently, Dahl et al. [5] and Zhang et al. [6] profiled the genome-wide dynamics of H3K4me3 along with other chromatin features during pre-implantation embryonic development. Dahl et al. [5] revealed that approximately 22% of the oocyte genome is associated with broad H3K4me3 domains that are negatively correlated with DNA methylation. They found that H3K4me3 becomes confined to TSS regions at the 2-cell stage. Zhang et al. [6] also observed a broad enrichment pattern of H3K4me3 in oocytes, but instead named this pattern as the non-canonical form of H3K4me3 (ncH3K4me3). Dahl et al. [5] and Zhang et al. [6] both highlighted that broad H3K4me3 in oocytes are not generally located near the TSS. In agreement with Liu et al. [7] and Benayoun et al. [3], they also demonstrate that the histone demethylases, *e.g.*, KDM5A and KDM5B, are required for shortening of broad H3K4me3. They further revealed that removal of broad H3K4me3 domains is required for normal zygotic genome activation. Notably, Liu et al. [7] also observed non-canonical flat H3K4me3 domains with low fold enrichment in oocytes, and reported that these domains are removed in the 2-cell stage, although they focused mainly on broad domains that were established during the pre-implantation development. Overall, these studies highlighted non-canonical broad H3K4me3 as a key player in the process of dramatic epigenetic reprograming in cells at pre-implantation developmental stages.

## Discussion

An intriguing mystery, however, is the biological implications of the non-canonical broad H3K4me3 domains observed specifically in oocytes. Zhang et al. [6] highlighted that the global occurrence of non-canonical broad H3K4me3 coincides with genome silencing from mature oocytes to the early 2-cell stage, raising a paradoxical possibility that it may contribute to gene repression. They demonstrated that over expression of histone demethylases leads to significant downregulation of H3K4me3 and reactivates transcription in a substantial portion of oocytes. One potential connection between non-canonical broad H3K4me3 and genome silencing, however, might be H3K4me3-specifc reader proteins expressed preferentially in oocytes. Given that canonical H3K4me3 in other cell types is well known to be associated with transcription initiation or elongation, it is a pressing need to unveil the molecular mechanisms underlying the role of non-canonical H3K4me3 as a repressive mark in oocytes.

There is also a possibility that a technical effect in data normalization might strengthen or weaken the observation of non-canonical broad H3K4me3 in oocytes. Given that non-canonical H3K4me3 in oocytes is broader than canonical H3K4me3 in other cell types, normalizing the total ChIP-Seq signal to be the same may result in lower density values for non-canonical H3K4me3 in oocytes. On the other hand, assuming that non-canonical broad H3K4me3 actually exists in other cell types too, when the total ChIP-Seq signals were normalized, the absence of canonical high-density signals in oocytes might have allowed the non-canonical low-density signals to be enhanced accordingly. As a result, the normalization effect might have caused non-canonical broad H3K4me3 to be observed in oocytes, but continue to be overlooked in other cell types. It is therefore important to test these two possibilities in future studies using external spike-in sequences as a control for low-input ChIP-Seq or ChIP-PCR.

## Competing interests

The authors declare that they have no competing interests.
